# Cicada Endosymbionts Have tRNAs That Are Correctly Processed Despite Having Genomes That Do Not Encode All of the tRNA Processing Machinery

**DOI:** 10.1128/mBio.01950-18

**Published:** 2019-06-18

**Authors:** James T. Van Leuven, Meng Mao, Denghui D. Xing, Gordon M. Bennett, John P. McCutcheon

**Affiliations:** a Division of Biological Sciences, University of Montana, Missoula, Montana, USA; b Department of Life and Environmental Sciences, University of California, Merced, Merced, California, USA; University of Hawaii at Manoa

**Keywords:** endosymbionts, evolutionary biology, genomics, RNAseq, symbiosis, tRNA maturation

## Abstract

Gene loss and genome reduction are defining characteristics of endosymbiotic bacteria. The most highly reduced endosymbiont genomes have lost numerous essential genes related to core cellular processes such as replication, transcription, and translation. Computational gene predictions performed for the genomes of the two bacterial symbionts of the cicada Diceroprocta semicincta, “*Candidatus* Hodgkinia cicadicola” (*Alphaproteobacteria*) and “*Ca*. Sulcia muelleri” (*Bacteroidetes*), have found only 26 and 16 tRNA genes and 15 and 10 aminoacyl tRNA synthetase genes, respectively. Furthermore, the original “*Ca*. Hodgkinia cicadicola” genome annotation was missing several essential genes involved in tRNA processing, such as those encoding RNase P and CCA tRNA nucleotidyltransferase as well as several RNA editing enzymes required for tRNA maturation. How these cicada endosymbionts perform basic translation-related processes remains unknown. Here, by sequencing eukaryotic mRNAs and total small RNAs, we show that the limited tRNA set predicted by computational annotation of “*Ca*. Sulcia muelleri” and “*Ca*. Hodgkinia cicadicola” is likely correct. Furthermore, we show that despite the absence of genes encoding tRNA processing activities in the symbiont genomes, symbiont tRNAs have correctly processed 5′ and 3′ ends and seem to undergo nucleotide modification. Surprisingly, we found that most “*Ca*. Hodgkinia cicadicola” and “*Ca*. Sulcia muelleri” tRNAs exist as tRNA halves. We hypothesize that “*Ca*. Sulcia muelleri” and “*Ca*. Hodgkinia cicadicola” tRNAs function in bacterial translation but require host-encoded enzymes to do so.

## Abstract (precis)

The smallest bacterial genomes, in the range of about 0.1 to 0.5 million base pairs, are commonly found in the nutritional endosymbionts of insects. These tiny genomes are missing genes that encode proteins and RNAs required for the translation of mRNAs, one of the most highly conserved and important cellular processes. In this study, we found that the bacterial endosymbionts of cicadas have genomes which encode incomplete tRNA sets and lack genes required for tRNA processing. Nevertheless, we found that endosymbiont tRNAs are correctly processed at their 5′ and 3′ ends and, surprisingly, that mostly exist as tRNA halves. We hypothesize that the cicada host must supply its symbionts with these missing tRNA processing activities.

## INTRODUCTION

The smallest and most gene-poor bacterial genomes are from the nutritional endosymbionts of sap-feeding insects (1, 2). These tiny genomes have lost even seemingly essential genes, such as those involved in DNA replication and translation (2). In terms of genome size and coding capacity, insect endosymbiont genomes span the gap between their free-living bacterial cousins that retain the genes for performing essential cell functions and organelles of symbiotic origin that have lost nearly all of their genes (that is, the mitochondria and plastids), particularly those involved in replication, transcription, and translation (2, 3). Insect endosymbiont genomes thus provide an opportunity to learn more about key adaptations enabling codependent and highly integrated endosymbioses but in associations that are younger and more labile than the classic cellular organelles.

**FIG 1:**
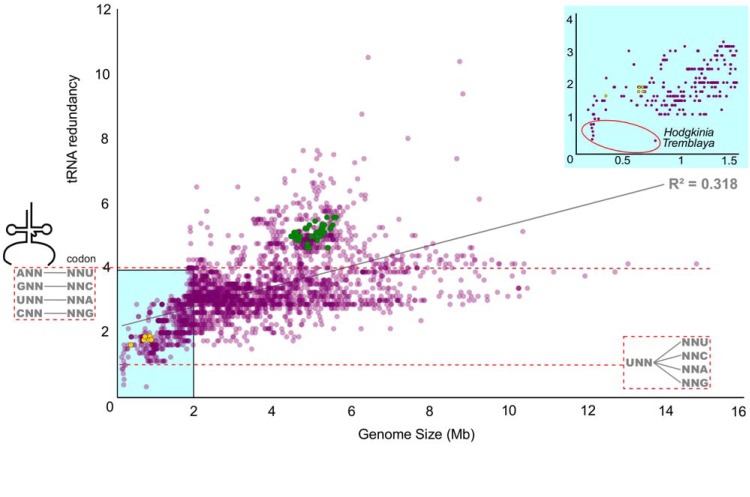
Genome size and tRNA redundancy are positively correlated. Each fully sequenced bacterial genome is shown as a dot (*n* = 2,761). tRNA redundancy data represent the number of total 4-box tRNA genes in a genome over the number of 4-box families. The red dashed line at *y* = 1 shows a limit where only one tRNA was found from each of the eight 4-box families. Below this limit, it is unclear if the organism has enough tRNAs for translation. The red dashed line at *y* = 4 shows one tRNA gene for each 4-box codon. Buchnera aphidicola and Escherichia coli are shown as yellow and green dots, respectively. Theoretically, all bacteria could function with a redundancy value of 1.

**FIG 2:**
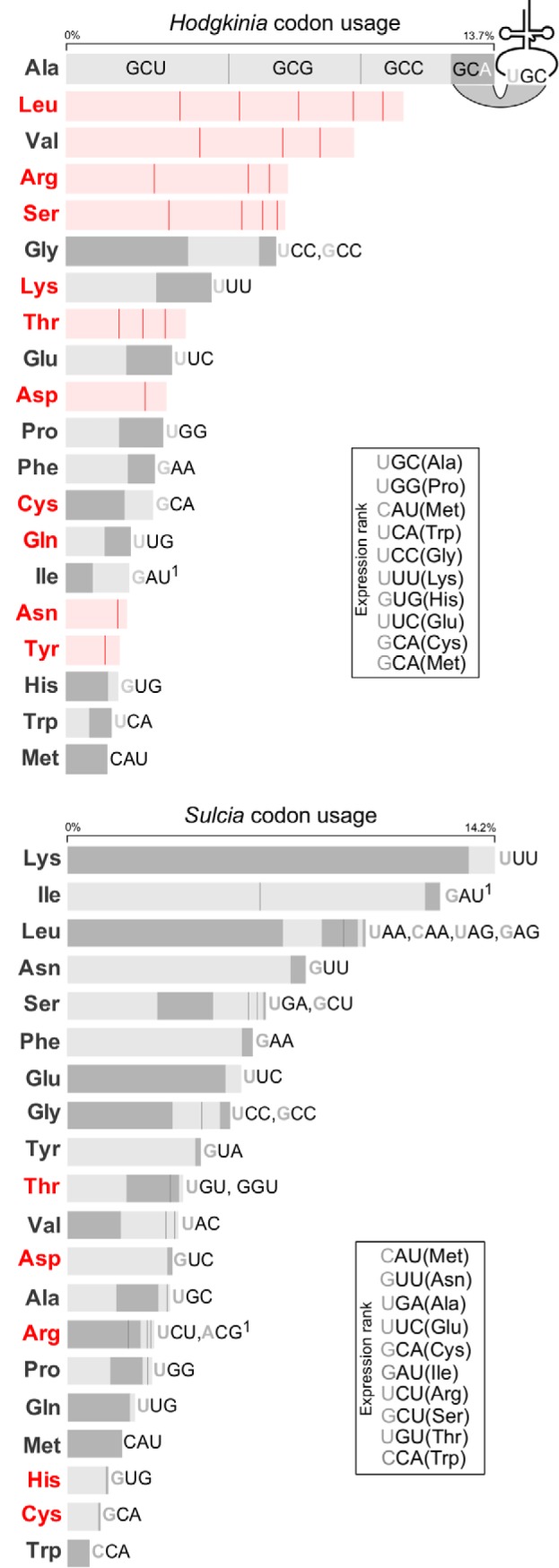
Codon usage and RNA expression in *Hodgkinia* and *Sulcia* genomes. Box sizes indicate codon frequencies of all protein-coding genes in *Hodgkinia* and *Sulcia*. Codons are ordered from highest to lowest usage; e.g., of the four alanine codons found in *Hodgkinia* protein-coding genes, GCU is used most frequently. The nucleotide sequences for *Hodgkinia* alanine codons (which make up 13.7% of the genome) are shown as an example; all others are omitted for simplicity of display. The presence of perfectly paired tRNAs is indicated by a dark gray box. Light gray fill indicates that a tRNA could possibly be used to translate the codon by N34 wobble. The anticodon sequence of each tRNA is shown to the right of its cognate codons and is written 5′ to 3′. N34 modifications that are likely needed for tRNA-codon pairing are indicated by a superscript “1.” A red-colored three-letter amino acid abbreviation indicates that the genome does not encode that aaRS. tRNA abundances determined by RNAseq are shown in the “Expression rank” boxes.

“*Candidatus* Hodgkinia cicadicola” (*Alphaproteobacteria*; hereafter *Hodgkinia*) and “*Ca*. Sulcia muelleri” (*Bacteroidetes*; hereafter *Sulcia*) have two of the smallest bacterial genomes published. *Sulcia* and *Hodgkinia* are obligate nutritional endosymbionts of many cicadas, where they work together to provide essential amino acids to their hosts (4). In the cicada Diceroprocta semicincta (Insecta: Hemiptera: Cicadoidea), the *Hodgkinia* genome is 143 kilobase pairs (kbp) in length and the *Sulcia* genome is 277 kbp. Only 16 tRNA genes and 10 of the 20 required aminoacyl-tRNA synthetase genes (aaRSs) were found in the *Hodgkinia* genome using computational methods (5). While the number of tRNA genes encoded in bacterial genomes is quite variable (Fig. 1), theoretical estimates predict that a minimum of ∼20 tRNAs are required to translate all 61 codons (6, 7). *Hodgkinia* is missing tRNA genes needed to decode leucine, valine, arginine, serine, threonine, aspartic acid, asparagine, and tyrosine codons (Fig. 2). The mealybug endosymbiont “*Candidatus* Tremblaya princeps” (here *Tremblaya*) also falls below the theoretical limit of 20, encoding only 8 to 12 tRNA genes and 0 or 1 aaRSs, depending on the strain (Fig. 1; see also Table S1 in the supplemental material) (8–11). However, *Tremblaya* is unusual in hosting its own intrabacterial endosymbiont, which may provide the missing tRNAs and aaRSs (9). There is no such explanation for the apparent lack of tRNA, aaRS, and tRNA processing genes in *Hodgkinia*.

10.1128/mBio.01950-18.3 TABLE S1 tRNA and aaRS genes in the smallest bacterial genomes. Shaded cells indicate that the aaRS gene listed in the leftmost column is missing. A superscript “1” indicates that the aaRS gene is heteromeric; a superscript “2” indicates a probable pseudogene. Download Table S1, DOCX file, 0.01 MB. Copyright © 2019 Van Leuven et al. 2019 Van Leuven et al. This content is distributed under the terms of the Creative Commons Attribution 4.0 International license.

Functional tRNAs are generated by a complex, multistep process that usually requires trimming off transcribed nucleotides that precede (5′ leader) and follow (3′ trailer) the predicted tRNA gene, posttranscriptional nucleotide editing at numerous positions, the addition of a terminal 3′ CCA, and aminoacylation of the mature tRNA to produce a molecule that is active on the ribosome. After transcription, 5′ leaders are trimmed by the nearly universal ribozyme RNase P (12, 13). The 3′ trailer is cleaved off by a combination of endonucleases and/or exonucleases (14), and if a terminal 3′ CCA is not encoded in the genome, one is added by a nucleotidyl transferase (15, 16). Finally, tRNA nucleotides are modified by a variety of enzymes at various conserved positions (17–19). These important modifications influence tRNA tertiary structure and interactions with cellular enzymes and proteins (17).

The original published computational annotation of the *Hodgkinia* genome from D. semicincta lacks most genes related to tRNA processing (5). It is missing the RNA (*rnpB*) and protein (*rnpA*) subunits of RNase P and the nucleases responsible for 3′ trailer trimming. *Hodgkinia* does not encode a CCA-adding enzyme, despite having only one tRNA gene with a genome-encoded terminal CCA. This *Hodgkinia* genome contains only three genes involved in tRNA editing (*mnmE*, *mnmA*, *and mnmG*), all of which are predicted to be involved in the conversion of uridine to 5-methylaminomethyl-2-thiouridine at U34 (5, 20). Because the genes encoding aaRSs and tRNA processing enzymes are large and typically highly conserved across life, it is unlikely that these proteins were missed in the original genome annotation.

The dramatic tRNA and aaRS gene loss observed in *Hodgkinia* is extremely rare in bacteria; only *Hodgkinia* and *Tremblaya* lack the theoretical minimum number of tRNAs and aaRS genes in their genomes (Fig. 1; see also Table S1). However, detection of tRNA genes in highly degraded endosymbiont genomes—particularly in mitochondrial genomes—is notoriously difficult (21, 22). Many mitochondrial tRNAs have unusual structures, in some cases missing entire d-loops, making them easy to miss by computational gene-finding algorithms unless they are specifically trained to find them (23). Similarly, the archaeal tRNA genes in the degenerate genome of *Nanoarchaeum* were initially missed because of the unusual way in that they are split and permuted (24, 25). We therefore reasoned that our initial computational annotation of the tRNAs of *Hodgkinia* (5) might be incomplete.

The apparent loss of these key genes in *Hodgkinia* raises several questions. Were *Hodgkinia* tRNAs and other small RNAs missed during computational gene prediction? For the tRNAs present on the genome, are their 5′ and 3′ ends correctly processed? Are *Hodgkinia* tRNAs modified only at the U34 wobble position as expected based on the gene content of the genome? Do host enzymes complement the missing symbiont genes? Here we address these questions by sequencing eukaryotic messenger and total small RNAs from the cicada species D. semicincta.

## RESULTS

### Endosymbiont tRNA genes are correctly annotated.

**FIG 3:**
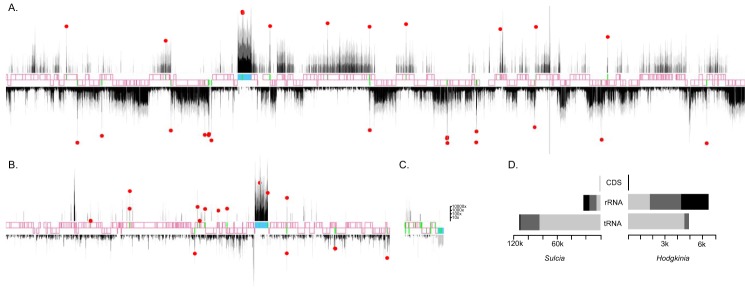
RNA expression patterns from the *Sulcia*, *Hodgkinia*, and mitochondrial genomes show relatively low levels of expression of *Hodgkinia* tRNAs. (A to C) Read depth plotted across the *Sulcia* (A), *Hodgkinia* (B), and mitochondrial (C) genomes. Protein-coding genes, rRNA genes, and tRNA genes on the sense and antisense strands are shown in pink, blue, and green, respectively. Red dots represent the highest read depth for each tRNA. Coverage depths for reads of lengths 18 to 47, 48 to 89, and 90 to 100 are shown in light gray, dark gray, and black, respectively, and data (drawn on a log10 scale) represent results after subtraction of background (median genic) coverage. (D) Median coverage depths for *Sulcia* (left) and *Hodgkinia* (right) for each gene category and read length. The bars are colored as described for panels A to C.

We sequenced small RNAs expressed in cicada bacteriome tissues to experimentally search for unannotated *Hodgkinia* and *Sulcia* tRNAs. Among 145,176,847 quality-filtered reads of length 18 to 90 nucleotides (nt), 15.6% and 28.4% map to the *Hodgkinia* and *Sulcia* genomes, respectively (see Table S2 in the supplemental material). While the mean levels of genome-wide read coverage of the *Sulcia* and *Hodgkinia* genomes were similar (224 reads/bp and 164 reads/bp, respectively), *Hodgkinia* tRNA expression levels were extremely variable (Fig. 3).

10.1128/mBio.01950-18.4 TABLE S2 The number of small RNA reads binned by size and mapped to full genomes and tRNA genes. A total of six barcoded libraries were made and sequenced. The pool comprising indices 1 to 4 contained the combined reads from libraries 1 to 4. Important percentages that are mentioned in the paper are shown in bold. Download Table S2, DOCX file, 0.02 MB. Copyright © 2019 Van Leuven et al. 2019 Van Leuven et al. This content is distributed under the terms of the Creative Commons Attribution 4.0 International license.

In *Sulcia*, we found that >99% of the reads mapped to tRNAs, transfer-messenger RNA (tmRNA), RNase P, and ribosomal RNAs (Table S2). The median depth of the reads mapping to the rest of the genome was 380×. To identify unannotated tRNAs, we manually inspected read coverage across the genome to identify regions with pronounced spikes in coverage. Only one of these high coverage spikes was found in an intergenic region (positions 75,798 to 75,839). All other spikes occurred within the bounds of annotated protein-coding sequences (CDSs). The spike at positions 75,798 to 75,839 corresponds to a Thr^GGT^ tRNA that was unannotated in the original *Sulcia* annotation due to a small assembly error in the published genome. We updated the *Sulcia* genome to reflect this change (NCBI reference sequence NC_012123.1; see Table S3 for primer sequences). In contrast to the tRNA reads, the reads contributing to CDS spikes did not have terminal CCAs or predicted folded structures that resembled tRNA (26). In summary, none of the spikes in coverage can be attributed to unannotated tRNAs.

10.1128/mBio.01950-18.5 TABLE S3 Primer sequences to amplify tRNAs from total RNA, genomic DNA, and finished library preparations. Download Table S3, DOCX file, 0.01 MB. Copyright © 2019 Van Leuven et al. 2019 Van Leuven et al. This content is distributed under the terms of the Creative Commons Attribution 4.0 International license.

In *Hodgkinia*, we found high expression of predicted tRNAs, ribosomal RNAs, and the 5′ ends of CDSs. Our analysis uncovered previously unannotated RNase P and tmRNA genes (discussed below [“Discovery of unannotated RNase P and tmRNA genes in *Hodgkinia*” section]). Of the total of 16 tRNA genes of *Hodgkinia*, many are not expressed above background (Fig. 3; see also Table S4). tRNA genes Gly061 and Gly108 each have no full-length reads aligning to them, even when allowing for 5 to 8 mismatches to accommodate modified bases (Table S4).

10.1128/mBio.01950-18.6 TABLE S4 Number of reads mapping to each tRNA gene (plus 15-bp flanking sequence) determined using bowtie. The 18-90 SAM file was parsed for reads that map to the tRNA with nearly the perfect length and ending in CCA. Download Table S4, DOCX file, 0.02 MB. Copyright © 2019 Van Leuven et al. 2019 Van Leuven et al. This content is distributed under the terms of the Creative Commons Attribution 4.0 International license.

Mapping reads to endosymbiont genomes allowed us to characterize tRNA processing patterns and to uncover expression of unannotated genes but was not well suited for identifying spliced or otherwise unconventional RNAs, such as intron-containing tRNAs. Therefore, we collapsed identical reads of length 48 to 90 nt and searched these collapsed reads for sequences that contained predicted tRNA genes, that ended in CCA, or that partially matched the *Sulcia* or *Hodgkinia* genomes. Because the number of unique (or nearly unique) reads was very high, we chose a minimum coverage cutoff value of 100×. The only transcript we found belonging to *Sulcia* or *Hodgkinia* (Blast E value, <1E−25) that was not an annotated tRNA or tmRNA was the previously unannotated *Sulcia* Thr^GGT^ transcript described above. Given these data, we conclude that the computational tRNA predictions for *Hodgkinia* and *Sulcia* are correct and that these genomes do not encode complete sets of tRNAs.

### Most tRNAs are found as tRNA halves.

**FIG 4:**
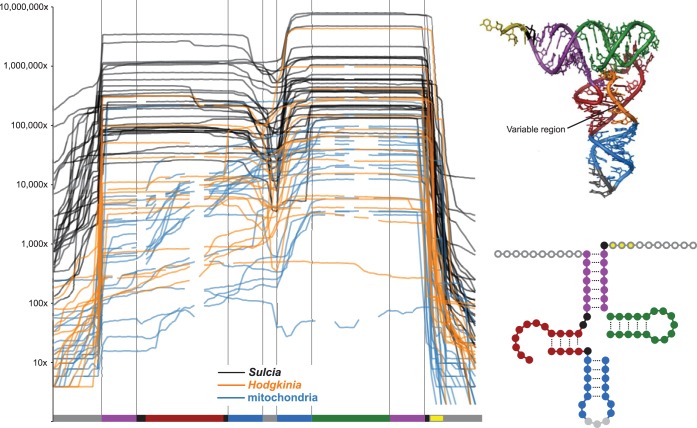
Dynamic range of tRNA half expression in *Sulcia* and *Hodgkinia*. Line graphs show read depth across each tRNA in *Hodgkinia* (orange), *Sulcia* (black), and the cicada mitochondria (blue). Because the tRNA lengths differed, only the bases present in all symbiont tRNA genes are shown in the structural diagrams of tRNAs. Mitochondrial tRNAs were often missing these regions, as indicated by gaps in the line graph. A total of 18 to 100 nucleotide reads were mapped for this figure.

Among 145,176,847 quality-filtered reads that were 18 to 90 nt in length, only 0.05% (74,651) and 4.8% (2,520,749) mapped in full length to *Hodgkinia* and *Sulcia* tRNAs, respectively (Table S2). Most reads were shorter than the predicted tRNA genes (Fig. 4; see also Table S4). The abundance of short reads could have been caused by RNA degradation, PCR bias toward short amplicons during library creation, or bona fide stable tRNA halves (27–30). Because our library creation protocol included reverse transcription (RT) after 5′ and 3′ RNA adapter ligation, these short reads are not likely due to reverse transcriptase failing to proceed through modified nucleotides, because these truncated reverse transcription products would not include both priming sites for PCR. To verify that the large number of tRNA halves that we found was not due to a bias against full-length but modified tRNAs, we created small RNA libraries where the RNA had been treated with the demethylating enzyme AlkB prior to reverse transcription (31, 32). This enzyme removes N^1^-methyladenosine (m^1^A), N^1^-methylguanosine (m^1^G), and N^3^-methylcytosine (m^3^C) modifications from tRNAs, and treatment of tRNAs with this enzyme has been shown to help recover more tRNAs by sequencing from some organisms (31, 32). We found that AlkB treatment had no effect on the length distribution or nucleotide modification rates of reads mapped to *Sulcia*, *Hodgkinia*, and mitochondrial DNA (mtDNA) (see Fig. S1 in the supplemental material; see also Table S2), suggesting that these methylation tRNA modifications were not affecting our results in any meaningful way. We therefore conclude that there are large amounts of either tRNA degradation products or stable tRNA halves present in the adult cicada bacteriome.

10.1128/mBio.01950-18.1 FIG S1 Read length distributions are very similar for AlkB-treated and untreated RNAs. The histogram shows the fraction of total reads at each length. Download FIG S1, EPS file, 0.5 MB. Copyright © 2019 Van Leuven et al. 2019 Van Leuven et al. This content is distributed under the terms of the Creative Commons Attribution 4.0 International license.

### Discovery of unannotated RNase P and tmRNA genes in *Hodgkinia*.

By aligning small RNA reads to the *Hodgkinia* genome, we found expression of previously unannotated RNase P (*rnpB*) and tmRNA (*ssrA*) genes at positions 25,448 to 25,794 and 92,713 to 93,140 in the *Hodgkinia* NC_012960.1 genome. Given that the 5′ ends of *Hodgkinia* tRNAs are correctly processed (see the next section), and that we cannot find any other RNA nucleases in *Hodgkinia*, it seems likely that this RNase P is responsible for the observed 5′ tRNA processing. The permuted *Hodgkinia* tmRNA is coded for in the reverse direction, on the antisense strand (Fig. S2). All components typically conserved in tmRNA structures can be found in the proposed tmRNA gene; however, the peptide tag does not end in the canonical YALAA sequence (33, 34). The coding RNA and acceptor RNAs are separated by a 129-nt intervening sequence containing complementary sequences needed for folding, and very few reads map to this region. Polymorphic sites indicate CCA addition at the 3′ end of both the coding and acceptor RNAs, further supporting the idea of the likely functionality of tmRNAs (35), especially given the presence of the gene for its protein ligand (*smpB*) in at least two *Hodgkinia* genomes from different cicada species (36). We also observed reads of various lengths at both the 5′ and 3′ ends of the tmRNA gene, indicating that end trimming probably occurs.

10.1128/mBio.01950-18.2 FIG S2 The proposed tmRNA gene in *Hodgkinia* lies between genes for EF-1 alpha and 16S rRNA. The direction of transcription is indicated by an arrow. EF-1 alpha and 16S are encoded on the sense strand. The tmRNA and *Hodgkinia*_127 are encoded on the antisense strand. Two small RNA transcripts with high coverage were identified as shown by separate arrows. Read depth across the tmRNA gene ranged from 0× to 50,000×. Coordinates of tmRNA features are shown for the coding and acceptor RNAs. Download FIG S2, EPS file, 0.2 MB. Copyright © 2019 Van Leuven et al. 2019 Van Leuven et al. This content is distributed under the terms of the Creative Commons Attribution 4.0 International license.

### The 5′ end processing of *Hodgkinia* and *Sulcia* tRNAs.

Many reads aligning to *Hodgkinia* and *Sulcia* tRNA genes extend past the predicted gene boundary, suggesting that they are transcribed with 5′ leaders and that these extra nucleotides are trimmed off (Fig. 4). We previously predicted the presence of the RNA moiety of RNase P RNA in *Sulcia* (5), and we now predict the presence of this gene in *Hodgkinia* as described above. To help verify the activity of both the *Hodgkinia* and *Sulcia* RNase P RNAs, we created two pools of Illumina-compatible small RNA libraries. In the creation of both libraries, RNA adapter sequences are ligated directly to the small RNA pools at the 5′ and 3′ ends. Adapter ligation can be blocked by either a triphosphorylated or diphosphorylated 5′ RNA end, but a functional RNase P generates 5′ monophosphate ends which are and active for ligation (80). By splitting one pool of small RNAs into two groups, one treated with tobacco acid pyrophosphatase (TAP), which would generate 5′ monophosphates, and one untreated, we tested the 5′ processed state of bacteriome tRNAs (13, 19, 37). In both *Hodgkinia* and *Sulcia*, we found no difference between the tRNA sets from each library (Spearman's rank correlation, *P* < 0.005; Table S5), suggesting that the 5′ ends of tRNAs are monophosphorylated in the cicada bacteriome. This is consistent with the presence of an active RNase P enzyme in both bacterial endosymbionts.

10.1128/mBio.01950-18.7 TABLE S5 No difference was found between TAP-treated and untreated libraries by Spearman's rank correlation and analysis of variance (ANOVA), indicating that the 5' end of *Hodgkinia* and that of *Sulcia* tRNAs were properly processed. Spearman's rank shows a significant correlation between tRNA expression of TAP-treated and untreated samples. ANOVA results showed no significant difference between the tRNA relative abundances of the treated and untreated samples. Relative abundance data represent the number of reads corresponding to each tRNA in comparison to the total number of reads assigned to all tRNAs in the sample. Categories for “Organism” include *Sulcia*, *Hodgkinia*, mitochondrial (*D. semicincta*), and other (unidentified). Significant *F* values for the ANOVA results are as follows: *F*(0.01) = 3.14; *F*(0.05) = 4.95. An edgeR analysis was performed for all four small RNA samples and for three read size ranges as follows: index 1, TAP-treated 2010 sample; index 2, 2010 sample; index 3, 2012 sample; index 4, 2012 sample; index 5, 2018 AlkB-treated library; index 6, 2018 untreated. The numbers of differentially expressed tRNA genes (among 66 total endosymbiont and mitochondrial genes) are shown. Download Table S5, DOCX file, 0.01 MB. Copyright © 2019 Van Leuven et al. 2019 Van Leuven et al. This content is distributed under the terms of the Creative Commons Attribution 4.0 International license.

### The 3′ ends of *Hodgkinia* and *Sulcia* tRNAs are correctly processed.

**FIG 5:**
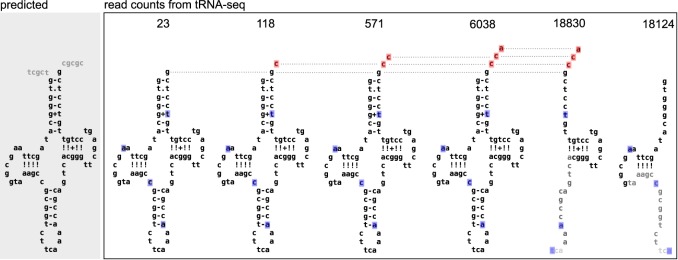
tRNA processing occurs in a stepwise manner, but full-length tRNAs comprise a small minority of the total reads. The majority of reads mapping to the *Hodgkinia* tRNA Trp062 gene (51,683) mapped to one of the secondary structures shown. Polymorphic sites (>2%) are shown in blue (RNA modifications) or red (CCA addition). tRNA halves are colored to indicate common sites of RNA degradation, where black letters indicate the highest read depth.

The processing of tRNA 3′ ends is more complicated than the processing of 5′ ends. If a 3′ CCA is not encoded on the genome (the majority of *Sulcia* and *Hodgkinia* tRNA genes do not have encoded CCA ends), one must be added by a CCA transferase enzyme after the 3′ trailer sequence is removed through processing by various RNA nucleases. Consistent with the presence of 3′ trailer sequences, we found reads extending past the predicted 3′ boundary of *Hodgkinia* and *Sulcia* tRNAs (Fig. 4; see also Fig. 5). *Sulcia* contains a putative RNase (ACU52822.1) that could potentially process the 3′ trailer, although the gene is most similar to that corresponding to RNase Y, which is involved in mRNA decay (38). *Hodgkinia* contains no such RNA nuclease candidates. We also observed reads ending in C, CC, and CCA that mapped to *Sulcia* and *Hodgkinia* tRNA genes, indicating that the nucleotides of the terminal CCA were added one at a time to the 3′ end of transcripts lacking 3′ trailers (Fig. 5). *Sulcia* contains a tRNA CCA nucleotidyl transferase, but *Hodgkinia* does not. Our mRNA sequencing (mRNA-Seq) data show upregulation of a tRNA CCA nucleotidyl transferase encoded on the cicada genome in the bacteriome tissue, although we do not know if this enzyme is active on *Hodgkinia* tRNAs. However, in plants, mammals, and yeast, isoforms of this protein are localized to both the cytoplasm and to organelles (39–41), suggesting that this enzyme might be targeted to different cellular compartments in cicada cells.

### tRNA modification occurs in *Hodgkinia* and *Sulcia*.

**FIG 6:**
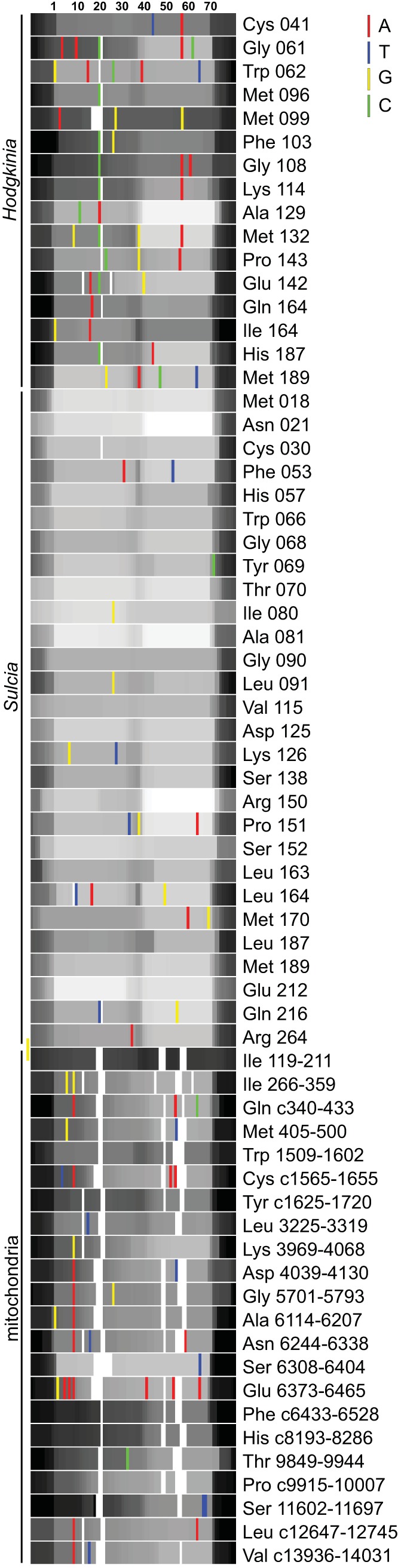
Expression level of individual tRNAs shown with polymorphic sites that have frequencies of greater than 2%. The per-base read depth was log transformed and is shown on a color scale corresponding to 0 to 255, making even large differences in expression levels difficult to distinguish by eye. Low expression is shown in black and high expression in white. Polymorphic sites are colored according to their genomic sequence. Ten bases of leader and trailer are shown as described for Fig. 5. Gaps are shown in white and are apparent primarily in mitochondrial RNAs (mtRNAs). See Table S2 for gene name descriptions.

The *Hodgkinia* genome encodes only three genes known to be involved in tRNA modification, all of which are predicted to act on U34: *mnmA*, *mnmG* (*gidA*), *and mnmE* (*trmE*) (5). MnmA catalyzes the 2-thiolation of U to s2U; MnmG and MnmE form a dimer that catalyzes the conversion of s2U to nm5s2U (20). The *Sulcia* genome carries these three genes, along with *truA* and *tilS* (5). TruA modifies U38-U40 to pseudouridine, and TilS converts C34 to I34, enabling the specific recognition of Met versus Ile anticodons (20). We found sequence polymorphisms in tRNA transcripts—which we interpret as potential base modifications (31, 42–44)—at several sites other than expected position 34 in *Hodgkinia* (positions 1, 4, 9, 11, 15, 16, 20, 23, 26, 27, 28, 37, 39, 40, 42, 43, 49, 57, 58, 62, 63, 64, and 68) and expected positions 34 and 38 to 40 in *Sulcia* (positions 6, 10, 11, 16, 20, 28, 31, 37, 49, 53, 54, 60, 64, 69, and 71) (Fig. 6; see also Table S6). Importantly, we observed no differences in modification patterns between AlkB-treated and untreated tRNAs (Table S6), suggesting that *Sulcia* and *Hodgkinia* do not have meaningful levels of m^1^A, m^1^G, or m^3^C modifications in their tRNAs. For a position to be called polymorphic, we required a read depth of at least 10× and greater than 2% polymorphism at the modified site. Interestingly, *Hodgkinia* tRNAs are more highly modified than *Sulcia* tRNAs in both the diversity of modification and the total number of tRNAs modified. Among the 16 tRNAs of *Hodgkinia*, 15 have at least one putatively modified site, versus 8 of 28 in *Sulcia* and 10 of 22 in the mitochondrial genome (Fig. 6; see also Table S6).

10.1128/mBio.01950-18.8 TABLE S6 tRNA modifications sorted by site. Those shown were present at a frequency of 2% or greater in at least one of the treatment types. The genome sequence, the read coverage, and the percentage mismatched at each putative modification size are shown. A superscript “a” indicates that the edit occurred on mismatched base pairs in the stem region; a superscript “b” indicates that tRNAs with high nucleotide similarity exist in the nuclear genome. A total of 48 to 90 nucleotide reads were used in mapping. Download Table S6, DOCX file, 0.03 MB. Copyright © 2019 Van Leuven et al. 2019 Van Leuven et al. This content is distributed under the terms of the Creative Commons Attribution 4.0 International license.

### Cicadas upregulate some aminoacyl tRNA synthetase transcripts in bacteriome tissues.

**FIG 7:**
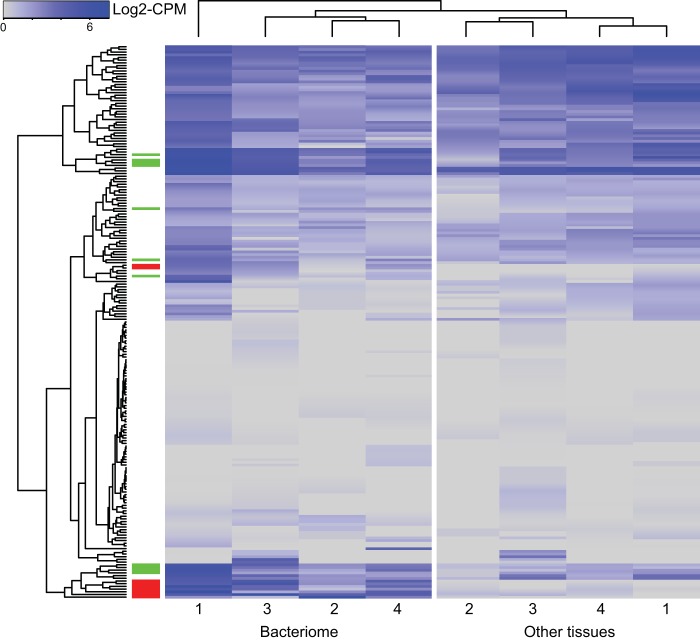
Host tRNA processing genes are rarely overexpressed in bacteriome tissues. Data representing expression of Trinity assembled genes whose Trinotate annotations involve tRNA processing are shown for bacteriome and nonbacteriome tissues for replicate insects 1 to 4. Genes and samples are clustered by Euclidian distance in R. Differentially expressed genes (edgeR, *P* > 0.05) are indicated by a red block in the leftmost column, and genes differentially expressed, but not significantly so, are indicated in green.

We sequenced cicada mRNAs from four insects in search of tRNA processing genes that could complement those missing from the *Hodgkinia* and *Sulcia* genomes. A total of 189,137 genes were assembled by Trinity from a combined total of 255,193,489 100-bp reads. The longest and mean contig lengths were 18,931 bp and 765 bp, respectively. Seventy-eight percent of the paired-end reads properly mapped in the transcriptome assembly, giving an overall Transrate assembly score of 0.21. A total of 21,651 nonredundant annotated protein-coding genes were found on the assembled contigs using Trinotate (Table S1). BUSCO orthologs were searched, and 75% of the core arthropod genes were found in our assembly, a typical value for hemipteran genomes (8). We used edgeR to identify 1,778 genes (Table S2) that were differentially expressed between bacteriome and insect tissues (false-discovery rate [FDR], ≤0.05). Among these 1,778 differentially expressed genes (DEGs), 1,211 had higher expression values in the bacteriome samples (Table S3). Most DEGs were not annotated by Trinotate and remained hypothetical proteins. Only nine genes upregulated in the bacteriome were annotated with a function involving tRNA maturation or charging, including five copies of d-tyrosyl-tRNA deacylase; two copies of an aminoacyl tRNA synthase complex-interacting multifunctional protein, tRNA uracil(54)-C(5)-methyltransferase B; and tRNA modification GTPase MnmE (Table S7). The gene expression profiles were wildly different between insects (Fig. 7), so we examined the most highly differentially expressed genes (top quartile) which had nonzero count-per-million values for at least three of the four replicates and were not identified as differentially expressed by edgeR. Among the 205 tRNA maturation genes identified by Trinotate, 9 not identified by edgeR were highly expressed in the bacteriome but not in other cicada tissues (Fig. 7). These genes are expressed at levels that are 50-fold to 2,586-fold higher in bacteriomes than in other tissues. Some of the genes highly expressed in bacteriomes are complementary with the functions missing from the *Hodgkinia* and *Sulcia* genomes, including the arginine, cysteine, and serine aaRSs (Table 1).

**TABLE 1:** Expression of cicada aaRS genes in complementing *Sulcia* and *Hodgkinia*

Genome	Expression (counts per million)^*a*^																			
	*alaS*	*asnS*	*aspS*	*argS*	*cysS*	*glnS*	*gltX*	*glyS*	*hisS*	*ileS*	*leuS*	*lysS*	*metG*	*pheS*	*proS*	*serS*	*thrS*	*trpS*	*tyrS*	*valS*
*Hodgkinia*	X						X	X	X	X			X	X	X			X		X
*Sulcia*	X	X				X	X	X		X	X	X	X	X	X	X		X	X	X
Cicada mitochondria	−0.1	1.2	0.5	0	16.8		−2.3		0	−4.7	−19.0		−0.4	−4.4	−0.7	−1.3		−2.2	−2.3	0.2
Cicada cytoplasm	3.0	−9.1	3.4	44	3.8	6.2	−8.7	−10.8	9.9	−20.9	4.2	−8.3	1.4	−0.6		12.9	29.1	6.1	−3.8	0.6

a The presence of aaRS genes in the *Hodgkinia* and *Sulcia* genomes is indicated by an “X.” Differential expression values (counts per million) of cicada aaRS genes are shown, where positive values indicate overexpression in the bacteriome (log2 counts per million in the bacteriome minus log2 counts per million in other tissues).

10.1128/mBio.01950-18.9 TABLE S7 List of transcripts that are involved in tRNA maturation and are upregulated in cicada bacteriocytes. Genes identified by edgeR (*P* < 0.05) and those highly expressed in bacteriocytes (*P* = nonsignificant [NS]) are listed. Gene expression values are given in counts per minute. Download Table S7, DOCX file, 0.02 MB. Copyright © 2019 Van Leuven et al. 2019 Van Leuven et al. This content is distributed under the terms of the Creative Commons Attribution 4.0 International license.

Five potential horizontal gene transfers (HGTs) from bacteria were identified in cicadas by Mao et al. (45) from the transcriptome assemblies reported here (Table S8), including a pectin lyase gene (*pel*) and two copies of a transcription regulatory gene (*yebC*), an AAA-ATPase gene, and a ribosome recycling factor gene (*frr*). None of these genes are predicted to play direct roles in tRNA production or maturation. Overall, the cicada genome seems to contain few genes from HGT that obviously complement lost endosymbiont genes (with the possible exception of *frr*), in contrast to several related sap-feeding insects (9, 45–48).

10.1128/mBio.01950-18.10 TABLE S8 Horizontally transferred genes identified in the cicada transcriptome. Gene expression values are given in counts per minute. Download Table S8, DOCX file, 0.01 MB. Copyright © 2019 Van Leuven et al. 2019 Van Leuven et al. This content is distributed under the terms of the Creative Commons Attribution 4.0 International license.

### Methodological caveats and complexities.

We found several unusual results while analyzing our data. First, we isolated highly abundant transcripts containing predicted tRNAs not belonging exclusively to *Hodgkinia*, *Sulcia*, or host tRNA genes. In these cases, the two halves of the transcript aligned to separate genomic locations of the endosymbionts or even to the genomes of separate organisms (half to *Sulcia* and half to *Hodgkinia*). In all cases, these were tRNA-like sequences that were joined near the anticodon. We could not amplify these RNAs from total RNA using gene-specific RT-PCR and thus concluded that they represent by-products of the RNA ligation steps of the library preparation. This serves as a cautionary result with respect to this method. Despite this, in all cases, true *Hodgkinia* and *Sulcia* tRNAs were also amplified, cloned, and sequenced as positive controls (see Table S3 for primer sequences). Second, our protocol may have selected against heavily modified tRNAs because of reverse transcription blocking (31, 49), although we tried to rule this possibility out by treating a library with the demodifying enzyme AlkB (Fig. S1). Since we found many tRNA sequences with a polymorphism(s) at conventionally modified sites, it is possible that reverse transcriptase can proceed over some modifications, consistent with previous findings (42–44, 49).

## DISCUSSION

### 5′ tRNA processing from the genomes of *Sulcia* and *Hodgkinia* can be explained, but 3′ processing cannot.

We did not computationally identify RNase P in our first *Hodgkinia* genome annotation (5), but here we report the presence of this gene in the *Hodgkinia* genome. While still apparently lacking the protein component (*rnpA*), trimming of 5′ tRNA leaders could still occur with the RNA component alone because it is a ribozyme (50). The 5′ processing of both *Hodgkinia* and *Sulcia* tRNAs can now be explained by normal cellular processes.

The way that *Sulcia* and *Hodgkinia* trim and process the 3′ ends of their tRNAs is less clear. Cleavage of the 3′ trailer from pre-tRNAs can be accomplished by a variety of redundant exonucleases and/or endonucleases (14), none of which are encoded in *Sulcia* or *Hodgkinia*. In Escherichia coli, RNase PH, RNase T, RNase D, and RNase II can all trim back the 3′ end of pre-tRNAs (14). In *Sulcia*, we identified an RNA nuclease with similarity to RNase Y. Although RNase Y is typically thought to initiate mRNA decay, it is also implicated in multiple RNA processing tasks (38). No such putative enzyme can be found in the *Hodgkinia* genome from D. semicincta, although a gene for the endoribonuclease YbeY is present in *Hodgkinia* genomes from other cicada species (51). After 3′ tRNA trailers are trimmed, a terminal CCA is added by a CCA-adding enzyme (19). This gene is conserved across all domains of life (52), including in *Sulcia*, but is apparently missing in *Hodgkinia*. We have identified transcripts belonging to *Hodgkinia* that have terminal C, CC, and CCAs. The presence of these variants indicates that *Hodgkinia* tRNAs are exposed to an active CCA-adding enzyme. One potential candidate is the host genome-encoded mitochondrial copy that is upregulated in bacteriome tissues (see Table S7 in the supplemental material). Mitochondrial CCA-adding enzymes are known to have broad specificity and functionality (39, 53) and so may work on *Hodgkinia* tRNAs if that are somehow trafficked to *Hodgkinia* cells by the host.

### Base modification, even if off-target and not biologically relevant, cannot be explained by the genes encoded in the *Sulcia* and *Hodgkinia* genomes.

Base modifications are essential for tRNA aminoacylation and codon recognition and have been well described in previous work (19), including the bacterial endosymbiont *Buchnera aphidicola* (43). tRNAs of *Buchnera* are posttranscriptionally modified at N37 and N34, but most of the observed modifications can be performed by enzymes encoded on the *Buchnera* genome (*miaA*, *miaB*, *rimN*, *trmD*, *tadA*, *queA*, *mnmA*, *mnmE*, *iscS*, *tilS*, and *gidA*). It is therefore a bit surprising that tRNA modifications were detected in *Hodgkinia* and *Sulcia* when the genes for these modification enzymes are not carried in their genomes. We note that *Hodgkinia* and mitochondrial tRNAs have more modifications in common than do those of *Hodgkinia* and *Sulcia* (Fig. 6; see also Table S6). Assuming that these “modifications” do not represent experimental artefacts of some sort, it seems possible that some of the host tRNA modification enzymes are active in *Hodgkinia* and *Sulcia*, although it is not clear if these modifications are biologically relevant.

### The potential problems with old cicadas.

Two results from this work suggest that using older cicadas—that is, cicadas that have emerged from the ground and are near the end of their long lives—for RNA work might be less than ideal. The first is our finding that both *Sulcia* tRNAs and *Hodgkinia* tRNAs exist primarily as tRNA halves in adult cicadas. While tRNA halves have been found in numerous other organisms and have been shown to have regulatory functions, they are often associated with organismal stress (27–30). It seems possible that harvesting cicadas as adults, near the end of their lives, might result in gene expression and RNA processing patterns associated with stress. Likewise, the mRNA expression values show wide swings in transcript abundance over our four biological replicates, and this also may be due to a breakdown of transcriptional regulation in elderly cicadas. It would be interesting to examine the state of cicada RNAs in actively growing nymphs in future work.

### Translational machinery is shared between host and organelle in eukaryotes.

The most gene-rich mitochondrial genomes of the Jakobid protists look very much like endosymbiont genomes and contain a full-fledged set of about 30 tRNA genes (54). In contrast, the most gene-poor mitochondrial genomes of some trypanosomatides and alveolates contain no tRNA genes (55). The range is similar in plastids, including 1 to 30 tRNA genes (56, 57). The sets of retained tRNAs in *Hodgkinia* and *Tremblaya* and in organelles overlap, but there are considerable differences (Table S1). Among 22 tRNA anticodon species in *Hodgkinia* and *Tremblaya* from the mealybug Planococcus citri, only trnA^UGC^, trnI^GAU^, trnM^CAU^, and trnF^UGC^ are present in both genomes (Table S1). The degrees of tRNA gene conservation of *Hodgkinia*, mitochondria, and plastids are strikingly similar (58, 59).

Unlike in insect endosymbionts, organellar aaRSs have been completely transferred to the nuclear genome (60–62). The processes involved in aaRS and tRNA import into organelles are complex and are reviewed elsewhere (60, 63, 64). The mechanisms for localizing charged tRNAs to the organelle are diverse and organism specific. In humans, for example, all aaRSs except GlnRS and LysRS are bacterially derived and targeted specifically to mitochondria to charge mitochondrially encoded tRNAs (65). In contrast, only 45 aaRS genes are expressed from the Arabidopsis thaliana genome; 21 are found only in the cytoplasm, 21 are dually targeted, 2 are chloroplast specific, and 1 is targeted to all three cellular compartments (81). The mitochondrial genomes of apicomplexans are missing tRNAs and aaRSs. They must import aminoacylated tRNAs that were charged in the cytoplasm (66). It is worth noting that the mitochondrial and plastid genomes of the plant A. thaliana contain 22 and 30 tRNA genes and yet cytosolic tRNAs are still imported (67). The import of seemingly unnecessary tRNAs occurs quite frequently, and in most cases, the role of redundant tRNAs in organelles is unknown (64). Our results strongly suggest that *Hodgkinia* and possibly *Sulcia* import tRNAs from their host cicada.

Despite the massive genetic integration of organelle with host (68, 69), most mitochondria and plastids retain genomes and thus some level of genetic autonomy. Gene retention patterns in the genomes of highly reduced bacterial symbionts also suggest a hurdle to giving up independence of some processes to their hosts, especially transcription, translation, and replication (2, 3). The *Hodgkinia* genome is lacking many genes involved in these processes, and our results reported here indicate that these missing functions are likely complemented by its host. While these results support the idea that some obligate symbioses may undergo major transitions to become a highly integrated unit (70), other recent data from other cicadas show that these integrated units are not inevitably stable, as sometimes *Hodgkinia* is lost and replaced by a new fungal symbiont (71).

## MATERIALS AND METHODS

### Sequencing small RNAs.

The bacteriomes of three wild-caught female D. semicincta cicadas collected around Tucson, AZ, in July 2010 and July 2012 were dissected in RNAlater. RNA was also extracted from a combination of brain, abdomen, and leg tissues as a nonbacteriome control. RNA was purified using a Roche High Pure miRNA isolation kit. Illumina libraries were made using a ScriptMiner small RNA-Seq library preparation kit (Epicenter). One sample was split into index 1 and index 2, and index 2 was treated with the supplied TAP enzyme to reduce the 5′ end to a monophosphate. The remaining two samples (index 3 and index 4) were processed without TAP treatment. In a separate experiment, RNA from one additional cicada was split into two samples, and one of the two was treated with the AlkB enzyme to remove m^1^A, m^1^G, and m^3^C nucleotide modifications. AlkB demethylation was performed as previously described (31). Briefly, about 15 pmol of small RNA was treated with a mixture of purified wild-type E. coli AlkB (500 pmol) and mutant AlkB (a D135S substitution [32]; 625 pmol) in 100 μl of demethylation buffer for 2 h at room temperature. The reaction was quenched by adding EDTA to reach a final concentration of 5 mM. Both the AlkB-treated RNA and the untreated RNA were purified using a Roche High Pure miRNA isolation kit. Reverse transcription was done with an adapter-specific primer using Superscript III (Invitrogen). Libraries were amplified for 15 rounds using FailSafe PCR enzyme mix (Epicenter) and the supplied primers. PCR bands that were 50 to 300 nt in size (including 113-nt adapters) were cut from an 8% polyacrylamide gel after staining with SYBR Safe (Invitrogen). Gel cutouts were shredded using 0.5-ml tubes with needle holes in the bottom and eluted in 300 μl 0.5 M ammonium acetate for 3.5 h at 37°C, and the contents were separated using a 0.22-μm-pore-size sterile filter. DNA was purified by isopropanol precipitation. A total of 226,712,931 100-nt single-end reads were generated on three HiSeq lanes at the Vincent J. Coates Genomics Sequencing Laboratory of the University of California (UC), Berkeley.

### Transcriptome sequencing (RNAseq) read processing for small RNAs.

Adapter sequences were trimmed using Cutadapt version 1.0 (-a AGATCGGAAGAGCACACGTCTGAACTCCAGTCAC -g AATGATACGGCGACCACCGACAGGT TCAGAGTTCTACAGTCCGACGATC -O 7) (72) and quality filtered (fastq_quality_filter -q 20 -p 90) using FASTX-Toolkit version 0.0.12 (http://hannonlab.cshl.edu/fastx_toolkit/). Reads shorter than 18 nt were discarded because identical matches of up to 16 nt in length can be found between different symbiont tRNA genes. Reads shorter than 48 nt were assumed to be too short to make a functional tRNA (73). Reads were mapped to *Hodgkinia* and *Sulcia* genomes and tRNA genes using either bowtie-1.0.0 (–best –maqerr 150 –seedlen 18) or bwa-0.7.5 aln (-n 0.08 -i 2) (74, 75).

### *De novo* small RNA discovery.

Identical reads were compressed using FASTX-Toolkit (fastx-collapser) and aligned to *Hodgkinia* and *Sulcia* tRNA genes (blastall 2.2.25, blastn -E 1E−25). Sequences that did not align to known tRNA genes were aligned to the *Hodgkinia* and *Sulcia* full-genome sequences (blastn -e 1E−10). The remaining sequences that did not align to the bacterial genomes were considered cicada sequences, and tRNAs were predicted using tRNAscan-SE 1.21 and ARAGORN 1.2.34 (76, 77). Nearly identical sequences were grouped into contigs using CAP3 (78). Collapsed sequences with different anticodons, 5′ leaders, or 3′ trailers that assembled together in CAP3 were separated.

### Comparing TAP-treated libraries to untreated libraries.

Reads that were 20 to 100 nt and 70 to 100 nt in length were mapped to a multi-fasta file containing *Hodgkinia*, *Sulcia*, and mitochondrial tRNA genes plus 15 bp of genome sequence flanking the gene using bowtie-0.12.7 (-f -S -n 3). The orders of tRNA coverage were compared between indexes using Spearman rank correlation (see Table S5 in the supplemental material). Scripts from Trinity v20140717 packages align_and_estimate_abundance.pl, abundance_estimates_to_matrix.pl, run_DE_analysis.pl, and analyze_diff_expr.pl were used to compare differential transcription levels (with parameters –SS_lib_type F –est_method RSEM –aln_method bowtie –seedlen 18 –maqerr 150 –best). Using separate *de novo* approaches for library 1 and library 2 (with 48-nt to 100-nt reads), we normalized tRNA coverage (number of reads per tRNA/total number of reads mapping to all tRNAs). A ratio representing the difference between library 1 coverage and library 2 coverage was calculated for each tRNA. For all values less than zero, the inverse was taken and multiplied by −1. In this way, we tried to capture the relative differences in expression levels for all tRNAs from all organisms. These data were tabulated such that the source organism, paired amino acid types, anticodon sequence, and relative expression changes for every tRNA were used as inputs in a linear model in R (Table S5).

### mRNAseq.

Reads from isolate SRR952383 were pooled with reads from an additional three cicadas and assembled using Trinity v2.1.1 with kmer_ length = 25 and min_contig_length = 48 (79). Assembled transcripts matching *Sulcia* and *Hodgkinia* were removed using bwa-mem v07.12. The remaining transcripts were annotated using Trinotate v2.0.2. Potential horizontally transferred genes (HTGs) were identified from the cicada transcripts with BLASTP searches against the bacterial nr database and verified against known HTGs from the leafhopper Macrosteles quadrilineatus (45), since none currently exists for D. semicincta. Among the 398,377 Trinotate annotated gene isoforms, 115,253 resulted in blast hits in the Swiss-Prot, TrEMBL, or EggNOG databases. A total of 1,284 contaminated transcripts were removed from the Trinity assembly and Trinotate annotation list following identification during submission to NCBI’s transcript sequence archive (TSA). Genes involved in tRNA processing were identified by searching for “tRNA” or “tRNA” in the Trinotate annotation. Expression values were analyzed and visualized in R using the edgeR, trinotateR, and ggplots2 packages.

### Cloning and sequencing of specific tRNAs.

tRNAs of interest were reverse transcribed using tRNA-specific primers (SuperScript III), amplified (NEB OneTaq), and cloned (Invitrogen TOPO TA cloning kit) using standard procedures. Plasmids were purified (Omega Plasmid Mini) and sequenced with the standard M13F primer.

### Bioinformatics.

Complete bacterial genome sequences were downloaded from ftp://ftp.ncbi.nlm.nih.gov/genomes/Bacteria/all.fna.tar.gz. Chromosomal sequences were searched for tRNA genes using tRNAscan-SE 1.21 and the bacterial model (77). tRNA redundancy was simply calculated by dividing the number of 4-box and 6-box family tRNA genes by the number of 4-box and 6-box families.

### Data availability.

Trinotate annotations for all transcripts assembled using Trinity are available at https://doi.org/10.6084/m9.figshare.6687089.v1.

Data corresponding to the expression levels of gene-level transcripts that were differentially expressed between bacteriome and other cicada tissues, shown as log2 fold change, are available at https://doi.org/10.6084/m9.figshare.6687089.v1.

Trinotate annotations for transcripts that are expressed at higher levels in cicada bacteriome tissues than in other cicada tissues are available at https://doi.org/10.6084/m9.figshare.6687089.v1.

Raw Illumina reads are available in NCBI's SRA database for the small RNA (SRR5081152 to 5081157) and transcriptome (SRS470226 and SRR5060328 to 5060333) data sets. Assembled transcript sequences are available from the NCBI TSA database (GGPH00000000.1).
