# Supplementary material for: Cicada Endosymbionts Have tRNAs That Are Correctly Processed Despite Having Genomes That Do Not Encode All of the tRNA Processing Machinery
Source: mBio. 2019 Jun 18;10(3):e01950-18. doi: 10.1128/mBio.01950-18 (PMC6581868; doi:10.1128/mBio.01950-18)
Supplement: TABLE S1 [file mBio.01950-18-st001.docx]

|  | **DICSEM** | **TETULN** | **TETUND1** | **TETUND2** | **MAGTRE** | **TPPAVE** | **TPPLON** | **TPPCIT** |
| --- | --- | --- | --- | --- | --- | --- | --- | --- |
| **valS** |  | UAC |  | UAC |  | GAC | GAC(2) | ^2^CAC |
| **ileS** | GAU | GAU |  | GAU | GAU | CAU,GAU | CAU | GAU |
| **proS** | UGG | UGG | UGG | UGG | UGG | UGG |  |  |
| **hisS** | GUG | GUG |  | GUG |  | GUG | GUG | ^2^GUG |
| **trpS** | UCA | UCA | UCA | UCA | UCA | CCA |  | CCA |
| **metG** | CAU(3) | CAU(3) | CAU(2) | CAU(2) | CAU | CAU(2) | CAU | CAU |
| **gltX** | UUC | UUC |  | UUG |  | UUC | UUC |  |
| **pheS^1^** | GAA | GAA | GAA | GAA |  | GAA | GAA | GAA |
| **pheT^1^** |  |  |  |  |  |  |  |  |
| **alaS** | UGC | UGC | ^2^GGC,UGC(2) | UGC |  | UGC | ACG,CCU | UGC |
| **glyS^1^** | UCC,GCC(2) | UCC,GCC | UCC | UCC,GCC | UCC,GCC | GCC,UCC | GCC,UCC |  |
| **glyQ^1^** |  |  |  |  |  |  |  |  |
| **serS** |  |  |  |  |  | CGA,GCU,GGA,UGA | ACU | UGA |
| **asnS** |  |  |  |  |  | GUU | GUU |  |
| **tryrS** |  |  |  |  |  | GUA |  |  |
| **glnS** | UUG | UUG | UUG | UUG | UUG | UUG |  | UUC |
| **lysS** | UUU | UUU |  | UUU | UUU | CUU,UUU | CUU(2) | CUU(3) |
| **leuS** |  |  |  |  |  | CAA,UAG | CAG |  |
| **argS** |  |  |  |  |  | ACG,UCU |  | ACG |
| **aspS** |  | GUC | GUC | GUC |  | GUC | GUC |  |
| **thrS** |  | UGU,GGU | UGU | UGU,GGU |  | CGU,GGU,UGU |  |  |
| **cysS** | GCA | GCA | GCA | GCA | GCA | GCA | GCA |  |
