# Supplementary material for: Cicada Endosymbionts Have tRNAs That Are Correctly Processed Despite Having Genomes That Do Not Encode All of the tRNA Processing Machinery
Source: mBio. 2019 Jun 18;10(3):e01950-18. doi: 10.1128/mBio.01950-18 (PMC6581868; doi:10.1128/mBio.01950-18)
Supplement: TABLE S2 [file mBio.01950-18-st002.docx]

|  | Index1  (TAP) | Index2  (untreated) | Index3  (untreated) | Index4  (untreated) | Index  1-4 pool | Index 5  (AlkB) | Index6  (untreated) |
| --- | --- | --- | --- | --- | --- | --- | --- |
| **Raw** | 77,189,680 | 19,096,461 | 47,564,122 | 82,862,668 | 226,712,931 | 19,339,002 | 25,634,628 |
| **Quality/length filtered** | 60,627,486 | 12,657,156 | 38,406,203 | 33,486,002 | 145,176,847 (100%) | 17,790,958 | 23,378,072 |
| **18-90nts** | 48,521,525 | 10,914,315 | 28,597,483 | 24,618,283 | 112,651,606 | 17,790,958 | 23,378,072 |
| Mapped to *Hodgkinia* | 7,484,021 (15.4%) | 1,565,097 (14.3%) | 7,677,511 (26.8%) | 5,903,119 (24%) | 22,629,748 **(15.6%)** | 3,590,664 (20.2%) | 4,503,594 (19.3%) |
| Mapped to *Sulcia* | 21,424,135 (44.2%) | 5,094,798 (46.7%) | 9,313,149 (32.6%) | 5,360,814 (21.8%) | 41,192,896 **(28.4%)** | 2,925,610 (16.4%) | 4,388,622 (18.8%) |
| Mapped to mitochondria | 261,693 (0.5%) | 83,062 (0.8%) | 57,994 (0.2%) | 77,055 (0.3%) | 479,804 (0.3%) | 36,038 (0.2%) | 61,383 (0.3%) |
| Mapped to *Hodgkinia* tRNAs | 2,545,941 (5.2%) | 635,453 (5.8%) | 101,749 (0.4%) | 386,447 (1.6%) | 3,669,590 (2.5%) | 50,669 (0.3%) | 70,377 (0.3%) |
| Mapped to *Sulcia* tRNAs | 15,582,990 (32.1%) | 3,489,686 (32%) | 1,732,283 (6.1%) | 2,646,973 (10.8%) | 23,451,932 (16.2%) | 527,570 (3%) | 782,797 (3.3%) |
| Mapped to mitochondrial tRNAs | 338,590 (0.7%) | 104,637 (1%) | 60,532 (0.2%) | 120,499 (0.5%) | 624,258 (0.4%) | 6,833 (0%) | 15,917 (0.1%) |
| **48-90nts** | 13,855,233 | 3,713,578 | 21,300,447 | 13,163,499 | 52,032,757 | 14,436,409 | 19,012,188 |
| Mapped to *Hodgkinia* | 3,706,879 (26.8%) | 24,261 (0.7%) | 7,151,011 (33.6%) | 4,812,614 (36.6%) | 15,694,765 (30.2%) | 3,332,790 (23.1%) | 3,982,785 (20.9%) |
| Mapped to *Sulcia* | 4,127,670 (29.8%) | 1,067,020 (28.7%) | 6,835,654 (32.1%) | 2,087,571 (15.9%) | 14,117,915 (27.1%) | 2,317,015 (16%) | 3,346,611 (17.6%) |
| Mapped to mitochondria | 81,003 (0.6%) | 24,261 (0.7%) | 47,331 (0.2%) | 38,985 (0.3%) | 191,580 (0.4%) | 26,871 (0.2%) | 42,840 (0.2%) |
| Mapped to *Hodgkinia* tRNAs | 13,254 (0.1%) | 4,277 (0.1%) | 17,712 (0.1%) | 39,408 (0.3%) | 74,651 **(0.5%)** | 2,442 (0%) | 5,549 (0%) |
| Mapped to *Sulcia* tRNAs | 885,324 (6.4%) | 229,957 (6.2%) | 691,271 (3.2%) | 714,197 (5.4%) | 2,520,749 **(4.8%)** | 114,893 (0.8%) | 226,045 (1.2%) |
| Mapped to mitochondrial tRNAs | 57,593 (0.4%) | 18,308 (0.5%) | 47,960 (0.2%) | 51,929 (0.4%) | 175,790 (0.34%) | 3,659 (0%) | 8,452 (0%) |
| **70-100nts** | 17,917,568 | 3,862,380 | 23,160,979 | 14,622,761 | 59,563,688 | 13,856,688 | 18,081,034 |
| Mapped to *Hodgkinia* | 3263,929 (18.2%) | 554,659 (14.4%) | 6,775,227 (29.3%) | 4,046,344 (27.7%) | 14,640,159 (24.6%) | 2,845,594 (20.5%) | 3,350,303 (18.5%) |
| Mapped to *Sulcia* | 9,572,979 (53.4%) | 1,591,156 (41.2%) | 10,075,795 (43.5%) | 6,042,490 (41.3%) | 27,282,420 (45.8%) | 1,766,416 (12.7%) | 2,310,574 (12.8%) |
| Mapped to mitochondria | 26,122 (0.1%) | 4,996 (0.1%) | 6,320 (0%) | 3,718 (0%) | 41,156 (0.1%) | 12,625 (0.1%) | 14,400 (0.1%) |
| Mapped to *Hodgkinia* tRNAs | 5,644 (0%) | 1,767 (0%) | 8,209 (0%) | 8,723 (0.1%) | 24,343 (0.04%) | 1,029 (0%) | 1,403 (0%) |
| Mapped to *Sulcia* tRNAs | 520,386 (2.9%) | 144,229 (3.7%) | 617,538 (2.7%) | 659,736 (4.5%) | 1,941,889 (3.3%) | 74,984 (0.5%) | 126,504 (0.7%) |
| Mapped to mitochondrial tRNAs | 674 (0%) | 128 (0%) | 321 (0%) | 558 (0%) | 1,681 (0%) | 1,308 (0%) | 1,682 (0%) |
