# Supplementary material for: Cicada Endosymbionts Have tRNAs That Are Correctly Processed Despite Having Genomes That Do Not Encode All of the tRNA Processing Machinery
Source: mBio. 2019 Jun 18;10(3):e01950-18. doi: 10.1128/mBio.01950-18 (PMC6581868; doi:10.1128/mBio.01950-18)
Supplement: TABLE S3 [file mBio.01950-18-st003.docx]

|  | **Forward primer 5' to 3'** | **Reverse primer 5' to 3'** |
| --- | --- | --- |
| Ala_129_*Hodgkinia* | GGGGCTGTAGCTCAATTGG | TGGAGCTAAGCGGACTCG |
| Cys_041_*Hodgkinia* | GGCTTCGTGGTATAGGGGT | GGCTTCGCTCAGACTCG |
| Thr_*Sulcia*_flanking | CCTGGACAATCTACATGAGCA | GGTAGAGCATCAGCCTTCCA |
| Split_tRNA_1 | AGAGTTGCCGGAGGGGTTAAC | TGGAGAATATCGGATTTGAACCG |
| Split_tRNA_2 | TATGGCAATAACCAAG | TGGAGAATATCGGATTTGAACCG |
| Split_tRNA_3 | GGTGGAGCAGTTGGTAGC | AGCTAAGCGGACTCGAACCGC |
| Split_tRNA_4 | GGTGAACGTAGCTCAATTGG | TGGAGCTAAGCGGACTCG |
| Split_tRNA_5 | GGATGTAGCGTAGGTTGG | CGGTACCGGGAATCGAACC |
| Split_tRNA_6 | CGCGGGGTGGAGCAGTTGG | CAACGGGGGCAGGAGTCG |
