# Supplementary material for: Cicada Endosymbionts Have tRNAs That Are Correctly Processed Despite Having Genomes That Do Not Encode All of the tRNA Processing Machinery
Source: mBio. 2019 Jun 18;10(3):e01950-18. doi: 10.1128/mBio.01950-18 (PMC6581868; doi:10.1128/mBio.01950-18)
Supplement: TABLE S4 [file mBio.01950-18-st004.docx]

|  | 18-90 | 48-90 | 70-100 | tRNA count |
| --- | --- | --- | --- | --- |
| SMDSEM_264_Arg | 171822 | 5010 | 4216 | 3522 |
| SMDSEM_216_Gln | 569993 | 13458 | 12207 | 291 |
| SMDSEM_212_Glu | 2345816 | 170866 | 163305 | 120854 |
| SMDSEM_189_Met | 390387 | 33319 | 29288 | 12978 |
| SMDSEM_187_Leu | 80814 | 1747 | 1746 | 542 |
| SMDSEM_170_Met | 110296 | 16461 | 15727 | 8919 |
| SMDSEM_164_Leu | 499915 | 54916 | 679 | 219 |
| SMDSEM_163_Leu | 142900 | 14619 | 14306 | 2513 |
| SMDSEM_152_Ser | 602352 | 96386 | 156066 | 35534 |
| SMDSEM_151_Pro | 1192993 | 2460 | 1995 | 1459 |
| SMDSEM_150_Arg | 4004740 | 56468 | 54731 | 36894 |
| SMDSEM_138_Ser | 232144 | 122460 | 4854 | 2366 |
| SMDSEM_126_Lys | 220987 | 16320 | 14855 | 6793 |
| SMDSEM_125_Asp | 557210 | 56272 | 51479 | 40892 |
| SMDSEM_115_Val | 92904 | 52027 | 50574 | 30918 |
| SMDSEM_091_Leu | 127084 | 28779 | 22692 | 17190 |
| SMDSEM_090_Gly | 50342 | 32083 | 29976 | 4065 |
| SMDSEM_081_Ala | 3605265 | 263269 | 122850 | 91939 |
| SMDSEM_080_Ile | 645895 | 159599 | 147330 | 95551 |
| SMDSEM_070_Thr | 1204147 | 175998 | 132236 | 68762 |
| SMDSEM_069_Tyr | 297423 | 21774 | 4172 | 1938 |
| SMDSEM_068_Gly | 249994 | 24795 | 22514 | 17837 |
| SMDSEM_066_Trp | 226724 | 38543 | 36839 | 30700 |
| SMDSEM_057_His | 272166 | 11285 | 9114 | 7518 |
| SMDSEM_053_Phe | 374054 | 2042 | 1844 | 1203 |
| SMDSEM_030_Cys | 219277 | 97275 | 96060 | 70828 |
| SMDSEM_021_Asn | 4102498 | 540031 | 335843 | 178660 |
| SMDSEM_018_Met | 861790 | 412487 | 404391 | 305158 |
| HCDSEM_189_Met | 306977 | 2522 | 1118 | 831 |
| HCDSEM_187_His | 220501 | 1654 | 373 | 250 |
| HCDSEM_164_Ile | 10009 | 65 | 8 | 3 |
| HCDSEM_163_Gln | 15973 | 103 | 48 | 30 |
| HCDSEM_143_Pro | 52512 | 6800 | 5805 | 9 |
| HCDSEM_142_Glu | 141333 | 37552 | 981 | 946 |
| HCDSEM_132_Met | 498517 | 330 | 108 | 78 |
| HCDSEM_129_Ala | 2254846 | 11053 | 7459 | 5591 |
| HCDSEM_114_Lys | 20565 | 112 | 58 | 13 |
| HCDSEM_108_Gly | 1859 | 60 | 3 | 0 |
| HCDSEM_103_Phe | 3561 | 156 | 4 | 3 |
| HCDSEM_099_Gly | 379 | 171 | 138 | 8 |
| HCDSEM_096_Met | 11795 | 1107 | 83 | 74 |
| HCDSEM_062_Trp | 51683 | 11390 | 7899 | 6754 |
| HCDSEM_061_Gly | 75091 | 388 | 1 | 0 |
| HCDSEM_041_Cys | 3989 | 1188 | 257 | 168 |
| DICSEMmt_Val_c(13936..14031) | 51028 | 6575 | 2 | 295 |
| DICSEMmt_Tyr_c(1625..1720) | 3553 | 107 | 10 | 5 |
| DICSEMmt_Trp_1509..1602 | 6858 | 75 | 4 | 28 |
| DICSEMmt_Thr_9849..9944 | 17606 | 692 | 51 | 9 |
| DICSEMmt_Ser_6308..6404 | 142889 | 114076 | 108 | 75887 |
| DICSEMmt_Ser_11602..11697 | 6822 | 23 | 41 | 1 |
| DICSEMmt_Pro_c(9915..10007) | 37229 | 6479 | 117 | 55 |
| DICSEMmt_Phe_c(6433..6528) | 2035 | 8 | 0 | 1 |
| DICSEMmt_Met_405..500 | 29118 | 1469 | 3 | 160 |
| DICSEMmt_Lys_3969..4068 | 80942 | 3324 | 129 | 83 |
| DICSEMmt_Leu_c(12647..12745) | 23661 | 4271 | 788 | 645 |
| DICSEMmt_Leu_3225..3319 | 15019 | 421 | 8 | 75 |
| DICSEMmt_Ile_266..359 | 36577 | 2388 | 4 | 162 |
| DICSEMmt_Ile_119..211 | 476 | 14 | 0 | 0 |
| DICSEMmt_His_c(8193..8286) | 1948 | 13 | 0 | 4 |
| DICSEMmt_Gly_5701..5793 | 8489 | 3501 | 85 | 351 |
| DICSEMmt_Glu_6373..6465 | 50668 | 17779 | 7 | 61 |
| DICSEMmt_Gln_c(340..433) | 34924 | 2491 | 323 | 479 |
| DICSEMmt_Cys_c(1565..1655) | 25463 | 3374 | 0 | 59 |
| DICSEMmt_Asp_4039..4130 | 20488 | 1068 | 0 | 32 |
| DICSEMmt_Asn_6244..6338 | 17953 | 3333 | 0 | 1370 |
| DICSEMmt_Ala_6114..6207 | 10512 | 4309 | 1 | 396 |
