# Supplementary material for: Cicada Endosymbionts Have tRNAs That Are Correctly Processed Despite Having Genomes That Do Not Encode All of the tRNA Processing Machinery
Source: mBio. 2019 Jun 18;10(3):e01950-18. doi: 10.1128/mBio.01950-18 (PMC6581868; doi:10.1128/mBio.01950-18)
Supplement: TABLE S5 [file mBio.01950-18-st005.docx]

| **Spearman's Rank** | | | | | |
| --- | --- | --- | --- | --- | --- |
|  | *Sulcia*  n (p=0.005 critical value) | *Hodgkinia*  n (p=0.005 critical value) | Rho value *Sulcia* | Rho value *Hodgkinia* |  |
| 20-100nts | 28 (0.496) | 16 (0.666) | **0.932** | **0.988** |  |
| 70-100nts | 28 (0.496) | 16 (0.666) | **0.943** | **0.962** |  |
|  |  |  |  |  |  |
| **ANOVA** |  |  |  |  |  |
|  | Df | Sum of squares | Mean of squares | F value | Pr (>F) |
| Organism | 4 | 8.17 | 2.04 | 0.4586 | 0.7658 |
| Amino acid | 45 | 193.69 | 4.30 | 0.9665 | 0.5423 |
| Anticodon | 16 | 59.13 | 3.70 | 0.8298 | 0.6482 |
| Organism:Amino acid | 3 | 4.51 | 1.50 | 0.3374 | 0.7984 |
| Organism:Anticodon | 35 | 208.40 | 5.95 | 1.3371 | 0.1529 |
| Anticodon:Amino acid | 3 | 5.61 | 1.87 | 0.4197 | 0.7394 |

**EdgeR**

|  | 18-90 nt | | | |  | 48-90 nt | | | |  | 70-100 nt | | | |
| --- | --- | --- | --- | --- | --- | --- | --- | --- | --- | --- | --- | --- | --- | --- |
| Index 1 | 0 |  |  |  |  | 0 |  |  |  |  | 0 |  |  |  |
| Index 2 | 0 | 0 |  |  |  | 0 | 0 |  |  |  | 0 | 0 |  |  |
| Index 3 | 20 | 20 | 0 |  |  | 18 | 16 | 0 |  |  | 20 | 20 | 0 |  |
| Index 4 | 11 | 11 | 3 | 0 |  | 11 | 9 | 4 | 0 |  | 11 | 11 | 3 | 0 |
