# Supplementary material for: Cicada Endosymbionts Have tRNAs That Are Correctly Processed Despite Having Genomes That Do Not Encode All of the tRNA Processing Machinery
Source: mBio. 2019 Jun 18;10(3):e01950-18. doi: 10.1128/mBio.01950-18 (PMC6581868; doi:10.1128/mBio.01950-18)
Supplement: TABLE S6 [file mBio.01950-18-st006.docx]

|  |  |  | **Pool** | | **Plus AlkB** | | **No AlkB** | |
| --- | --- | --- | --- | --- | --- | --- | --- | --- |
| Site | tRNA gene | Seq | Coverage | Mismatch | Coverage | Mismatch | Coverage | Mismatch |
| 1 | Mito_Ala_6114..6207 | g | 313 | 0.44 | 0 |  | 0 |  |
|  | Mito_Glu_6373..6465 | g | 95 | 0.06 | 4 | 1.00 | 1 | 1.00 |
|  | Hodgkinia_062 | g | 7521 | 0.00 | 1 | 0.00 | 11 | 0.18 |
|  | Hodgkinia_164 | g | 29 | 0.62^a^ | 0 |  | 0 |  |
| 4 | Mito_Cys_1565..1655 | t | 133 | 0.36^a^ | 0 |  | 0 |  |
|  | Hodgkinia_061 | a | 21 | 0.10 | 2 | 0.00 | 0 |  |
|  | Hodgkinia_099 | c | 129 | 0.12 | 0 |  | 2 | 1.00 |
| 5 | Mito_Glu_6373..6465 | a | 419 | 0.06 | 4 | 0.00 | 1 | 0.00 |
| 6 | Mito_Ile_266..359 | g | 375 | 0.75 | 0 |  | 0 |  |
|  | Mito_Met_405..500 | g | 328 | 0.12 | 0 |  | 1 | 0.00 |
|  | Sulcia_126 | g | 15707 | 0.09 | 248 | 0.01 | 129 | 0.00 |
| 7 | Mito_Glu_6373..6465 | a | 476 | 0.05 | 4 | 0.00 | 1 | 0.00 |
| 9 | Mito_Ala_6114..6207 | a | 691 | 0.25 | 0 |  | 0 |  |
|  | Mito_Asn_6244..6338 | a | 2829 | 0.78 | 0 |  | 0 |  |
|  | Mito_Asp_4039..4130 | a | 85 | 0.35 | 0 |  | 0 |  |
|  | Mito_Cys_1565..1655 | a | 152 | 0.24 | 0 |  | 0 |  |
|  | Mito_Gln_340..433 | a | 686 | 0.17 | 66 | 0.00 | 49 | 0.00 |
|  | Mito_Glu_6373..6465 ^b^ | a | 499 | 0.36 | 5 | 0.20 | 2 | 1.00 |
|  | Mito_Gly_5701..5793 | a | 991 | 0.34 | 0 |  | 0 |  |
|  | Mito_Ile_266..359 | g | 609 | 0.06 | 0 |  | 0 |  |
|  | Mito_Leu_12647..12745 | a | 995 | 0.39 | 0 |  | 0 |  |
|  | Mito_Lys_3969..4068 | g | 158 | 0.32 | 2 | 0.00 | 0 |  |
|  | Mito_Val_13936..14031^b^ | a | 756 | 0.58 | 1 | 0.00 | 0 |  |
|  | Hodgkinia_061 | a | 21 | 0.05 | 0 |  | 2 | 0.00 |
|  | Hodgkinia_132 | g | 127 | 0.17 | 8 | 0.00 | 2 | 0.00 |
| 10 | Sulcia_164 | t | 498 | 0.00 | 11 | 0.00 | 24 | 0.08 |
| 11 | Hodgkinia_129 | c | 7676 | 0.00 | 57 | 0.04 | 29 | 0.14 |
|  | Sulcia_164 | g | 500 | 0.00 | 11 | 0.09 | 22 | 0.00 |
| 15 | Mito_Val_13936..14031 | t | 5697 | 0.10 | 1 | 1.00 | 0 |  |
|  | Hodgkinia_061 | g | 28 | 0.79 | 2 | 1.00 | 0 |  |
|  | Hodgkinia_062 | a | 7959 | 0.00 | 3 | 0.00 | 14 | 0.07 |
|  | Hodgkinia_142 | a | 1261 | 0.89 | 0 |  | 0 |  |
| 16 | Mito_Leu_3225..3319 | t | 273 | 0.14 | 0 |  | 1 | 1.00 |
|  | Mito_Asn_6244..6338 | t | 3144 | 0.46 | 0 |  | 0 |  |
|  | Hodgkinia_164 | a | 48 | 0.56 | 0 |  | 0 |  |
|  | Sulcia_164 | a | 518 | 0.00 | 24 | 0.00 | 24 | 0.08 |
| 20 | Hodgkinia_061 | c | 39 | 0.26 | 2 | 0.00 | 0 |  |
|  | Hodgkinia_096 | c | 257 | 0.30 | 0 |  | 0 |  |
|  | Hodgkinia_103 | c | 54 | 0.06 | 0 |  | 0 |  |
|  | Hodgkinia_108 | c | 23 | 0.78 | 0 |  | 2 | 1.00 |
|  | Hodgkinia_114 | c | 74 | 0.11 | 8 | 0.00 | 0 |  |
|  | Hodgkinia_129 | a | 9737 | 0.00 | 68 | 0.00 | 35 | 0.06 |
|  | Hodgkinia_132 | c | 150 | 0.62 | 8 | 0.00 | 2 | 0.00 |
|  | Hodgkinia_142 | c | 1428 | 0.95 | 1 | 0.00 | 0 |  |
|  | Hodgkinia_163 | c | 77 | 0.70 | 0 |  | 0 |  |
|  | Hodgkinia_187 | c | 787 | 0.35 | 2 | 0.00 | 2 | 0.00 |
|  | Sulcia_216 | t | 13128 | 0.00 | 18 | 0.06 | 31 | 0.00 |
| 23 | Hodgkinia_143 | c | 6375 | 0.00 | 17 | 0.12 | 5 | 0.00 |
|  | Hodgkinia_189 | g | 2312 | 0.21^a^ | 26 | 1.00^a^ | 15 | 1.00^a^ |
| 26 | Mito_Gly_5701..5793 | g | 3429 | 0.37 | 0 |  | 0 |  |
|  | Hodgkinia_062 | c | 8520 | 0.00 | 21 | 0.00 | 20 | 0.05 |
|  | Hodgkinia_103 | g | 124 | 0.69 | 1 | 1.00 | 0 |  |
| 27 | Hodgkinia_099 | c | 150 | 0.19 | 0 |  | 2 | 1.00 |
| 28 | Hodgkinia_189 | a | 2403 | 0.00 | 25 | 0.04 | 17 | 0.12 |
|  | Sulcia_126 | t | 16239 | 0.00 | 264 | 0.00 | 135 | 0.06 |
| 31 | Sulcia_053 | a | 2006 | 0.00 | 25 | 0.08 | 11 | 0.00 |
| 32 | Mito_Thr_9849..9944 | c | 658 | 0.09 | 0 |  | 0 |  |
| 34 | Sulcia_151 | t | 2374 | 0.00 | 67 | 0.06 | 32 | 0.13 |
|  | Sulcia_264 | a | 4968 | 0.08 | 175 | 0.00 | 188 | 0.01 |
| 37 | Hodgkinia_132 | g | 315 | 0.09 | 7 | 0.00 | 2 | 0.00 |
|  | Hodgkinia_143 | g | 6572 | 0.07 | 3 | 0.00 | 2 | 0.00 |
|  | Hodgkinia_189 | g | 2408 | 0.42 | 6 | 0.33 | 6 | 0.33 |
|  | Sulcia_151 | g | 2358 | 0.09 | 49 | 0.00 | 19 | 0.00 |
| 39 | Hodgkinia_062 | a | 8728 | 0.00 | 36 | 0.00 | 13 | 0.15 |
| 40 | Hodgkinia_142 | g | 4221 | 0.00 | 23 | 0.09 | 7 | 0.00 |
| 41 | Mito_Glu_6373..6465 | a | 17251 | 0.00 | 25 | 0.00 | 14 | 0.07 |
| 42 | Hodgkinia_041 | t | 814 | 0.46^a^ | 2 | 1.00^a^ | 3 | 1.00^a^ |
| 43 | Hodgkinia_187 | a | 1599 | 0.98 | 7 | 1.00 | 3 | 1.00 |
| 49 | Hodgkinia_189 | c | 2381 | 0.20 | 6 | 1.00 | 6 | 1.00 |
|  | Sulcia_164 | g | 5876 | 0.02 | 29 | 0.00 | 20 | 0.05 |
| 53 | Sulcia_216 | g | 13351 | 0.00 | 46 | 0.00 | 25 | 0.08 |
| 54 | Mito_Asp_4039..4130 | a | 885 | 0.27 | 0 |  | 0 |  |
|  | Mito_Cys_1565..1655 | c | 1591 | 0.17 | 2 | 1.00 | 0 |  |
|  | Sulcia_053 | t | 1928 | 0.00 | 23 | 0.09 | 2 | 0.00 |
| 55 | Mito_Met_405..500 | t | 1156 | 0.50 | 0 |  | 0 |  |
| 56 | Mito_Cys_1565..1655 | a | 1590 | 0.17 | 2 | 1.00 | 0 |  |
|  | Mito_Glu_6373..6465 | a | 17337 | 0.76 | 22 | 1.00 | 12 | 1.00 |
| 57 | Mito_Gln_340..433 | a | 2124 | 0.12 | 66 | 1.00 | 48 | 1.00 |
|  | Hodgkinia_143 | a | 6500 | 0.61 | 4 | 1.00 | 0 |  |
| 58 | Hodgkinia_041 | a | 752 | 0.43 | 3 | 0.00 | 4 | 0.00 |
|  | Hodgkinia_061 | a | 339 | 0.44 | 22 | 0.00 | 14 | 0.00 |
|  | Hodgkinia_108 | a | 39 | 0.15 | 2 | 0.00 | 2 | 0.00 |
|  | Hodgkinia_114 | a | 102 | 0.11 | 7 | 0.00 | 0 |  |
|  | Hodgkinia_163 | a | 102 | 0.55 | 0 |  | 0 |  |
| 60 | Sulcia_170 | t | 16377 | 0.00 | 68 | 0.00 | 36 | 0.06 |
| 62 | Mito_Asn_6244..6338 | a | 3175 | 0.43 | 0 |  | 0 |  |
|  | Hodgkinia_108 | a | 38 | 0.05 | 2 | 0.00 | 2 | 0.00 |
| 63 | Hodgkinia_061 | c | 337 | 0.00 | 20 | 0.05 | 14 | 0.00 |
|  | Hodgkinia_099 | g | 157 | 0.22 | 0 |  | 1 | 1.00 |
| 64 | Hodgkinia_189 | t | 2103 | 0.12 | 5 | 0.80 | 6 | 1.00 |
|  | Sulcia_151 | a | 2244 | 0.00 | 39 | 0.00 | 13 | 0.08 |
| 66 | Mito_Glu_6373..6465 | a | 17146 | 0.76 | 22 | 1.00 | 12 | 1.00 |
|  | Mito_Ser_6308..6404 | t | 109466 | 0.00 | 56 | 0.00 | 29 | 0.07 |
| 67 | Mito_Gln_340..433 | c | 2085 | 0.11 | 65 | 0.97 | 48 | 1.00 |
|  | Mito_Leu_12647..12745 | a | 3861 | 0.54 | 0 |  | 0 |  |
|  | Mito_Ser_11602..11697 | t | 20 | 0.05 | 0 |  | 0 |  |
| 68 | Mito_Ser_11602..11697 | t | 20 | 0.05 | 0 |  | 0 |  |
|  | Hodgkinia_062 | t | 8645 | 0.90^a^ | 47 | 1.00^a^ | 15 | 1.00^a^ |
| 69 | Sulcia_170 | g | 15857 | 0.00 | 53 | 0.11 | 33 | 0.00 |
| 71 | Sulcia_069 | c | 7711 | 0.00 | 871 | 0.16 | 268 | 0.38 |
