# Supplementary material for: Cicada Endosymbionts Have tRNAs That Are Correctly Processed Despite Having Genomes That Do Not Encode All of the tRNA Processing Machinery
Source: mBio. 2019 Jun 18;10(3):e01950-18. doi: 10.1128/mBio.01950-18 (PMC6581868; doi:10.1128/mBio.01950-18)
Supplement: TABLE S7 [file mBio.01950-18-st007.docx]

| Trinity | Product | 1b | 1o | 2b | 2o | 3b | 3o | 4b | 4o | pvalue |
| --- | --- | --- | --- | --- | --- | --- | --- | --- | --- | --- |
| DN38936_c1_g1 | Arginine--tRNA ligase, cytoplasmic | 129.9 | 5.5 | 6.2 | 1.3 | 34.3 | 3.4 | 20.8 | 5.0 | NS |
| DN38565_c0_g1 | Cysteine--tRNA ligase, cytoplasmic | 9.1 | 1.7 | 3.9 | 0.0 | 1.8 | 1.2 | 4.0 | 0.6 | NS |
| DN46824_c3_g1 | Cysteine--tRNA ligase, mitochondrial | 51.7 | 0.9 | 3.6 | 0.7 | 9.0 | 1.7 | 8.2 | 2.1 | NS |
| DN51624_c0_g1 | Serine--tRNA ligase, cytoplasmic | 54.9 | 3.7 | 1.2 | 0.3 | 2.1 | 2.7 | 2.7 | 2.7 | NS |
| DN36332_c0_g2 | Putative tRNA pseudouridine synthase Pus10 | 68.4 | 3.2 | 4.4 | 3.3 | 10.8 | 7.1 | 17.5 | 4.9 | NS |
| DN38950_c0_g1 | tRNA 2'-phosphotransferase 1 | 118.9 | 13.3 | 5.9 | 0.3 | 19.9 | 7.6 | 20.5 | 6.1 | NS |
| DN40306_c0_g1 | tRNA (guanine(10)-N2)-methyltransferase homolog | 15.1 | 1.1 | 0.6 | 2.6 | 2.3 | 1.4 | 6.2 | 1.8 | NS |
| DN43098_c0_g1 | Methionyl-tRNA formyltransferase, mitochondrial | 15.5 | 0.2 | 0.9 | 0.0 | 1.5 | 0.1 | 1.4 | 0.1 | NS |
| DN47148_c0_g2 | Queuine tRNA-ribosyltransferase subunit QTRTD1 homolog | 60.7 | 3.2 | 1.8 | 0.3 | 4.4 | 0.6 | 11.3 | 1.7 | NS |
| DN34313_c0_g1 | D-tyrosyl-tRNA(Tyr) deacylase 1 | 22.4 | 0.0 | 3.6 | 0.0 | 28.8 | 0.0 | 19.5 | 0.0 | 0.001 |
| DN41591_c0_g1 | tRNA modification GTPase MnmE | 3.3 | 0.0 | 119.8 | 0.0 | 0.7 | 0.1 | 6.3 | 0.0 | 0.012 |
| DN45267_c0_g1 | D-tyrosyl-tRNA(Tyr) deacylase 1 | 28.6 | 0.0 | 17.8 | 1.0 | 121.0 | 1.4 | 21.8 | 0.5 | 0.043 |
| DN45267_c2_g1 | D-tyrosyl-tRNA(Tyr) deacylase 1 | 20.7 | 0.0 | 4.2 | 0.0 | 48.3 | 0.0 | 8.7 | 0.0 | 0.002 |
| DN45267_c2_g2 | D-tyrosyl-tRNA(Tyr) deacylase 1 | 9.7 | 0.0 | 0.0 | 0.0 | 3.2 | 0.0 | 3.9 | 0.0 | 0.043 |
| DN47407_c0_g1 | tRNA (uracil(54)-C(5))-methyltransferase homolog-B | 160.2 | 0.3 | 4.4 | 0.3 | 1.6 | 0.5 | 21.7 | 0.8 | 0.031 |
| DN48812_c5_g14 | D-tyrosyl-tRNA(Tyr) deacylase | 31.0 | 0.0 | 0.6 | 0.0 | 13.6 | 0.0 | 4.2 | 0.0 | 0.006 |
| DN48899_c1_g2 | Aminoacyl tRNA synthase complex-interacting multifunctional protein 1 | 10.2 | 0.0 | 0.0 | 0.0 | 2.9 | 0.0 | 2.8 | 0.0 | 0.049 |
| DN52199_c1_g2 | Aminoacyl tRNA synthase complex-interacting multifunctional protein 1 | 93.4 | 0.1 | 1.2 | 0.0 | 13.0 | 0.2 | 12.6 | 0.0 | 0.011 |
