# Supplementary material for: Cicada Endosymbionts Have tRNAs That Are Correctly Processed Despite Having Genomes That Do Not Encode All of the tRNA Processing Machinery
Source: mBio. 2019 Jun 18;10(3):e01950-18. doi: 10.1128/mBio.01950-18 (PMC6581868; doi:10.1128/mBio.01950-18)
Supplement: TABLE S8 [file mBio.01950-18-st008.docx]

| Trinity | Gene | Product | 1b | 1o | 2b | 2o | 3b | 3o | 4b | 4o |
| --- | --- | --- | --- | --- | --- | --- | --- | --- | --- | --- |
| DN89433_c0_g1 | N/A | AAA-ATPase | 0.2 | 0 | 0 | 0 | 0 | 0 | 0.1 | 0 |
| DN22517_c1_g1 | *pel* | Pectin lyase | 0 | 0 | 0 | 0 | 0 | 1.4 | 0 | 1.4 |
| DN65002_c0_g1 | *yebC-1* | Transcription regulator | 9.9 | 0 | 0.4 | 0 | 9.9 | 0 | 2.6 | 0 |
| DN39720_c1_g1 | *yebC-2* |  | 12 | 0 | 0.4 | 0 | 1.5 | 0 | 5.4 | 0 |
| DN56768_c0_g1 | *frr* | Ribosome recycling factor | 0 | 0 | 0 | 0 | 3.6 | 4.1 | 0 | 0 |
